# Improving sleep after stroke: A randomised controlled trial of digital cognitive behavioural therapy for insomnia

**DOI:** 10.1111/jsr.13971

**Published:** 2023-07-05

**Authors:** Melanie K. Fleming, Tom Smejka, Ellie Macey, Ramon Luengo‐Fernandez, Alasdair L. Henry, Barbara Robinson, Simon D. Kyle, Colin A. Espie, Heidi Johansen‐Berg

**Affiliations:** ^1^ Wellcome Centre for Integrative Neuroimaging, FMRIB, Nuffield Department of Clinical Neurosciences University of Oxford Oxford UK; ^2^ Health Economics Research Centre, Nuffield Department of Population Health University of Oxford Oxford UK; ^3^ Big Health Ltd London UK; ^4^ Sir Jules Thorn Sleep & Circadian Neuroscience Institute, Nuffield Department of Clinical Neurosciences University of Oxford Oxford UK

**Keywords:** actigraphy, brain injury, cognitive behavioural therapy, cost‐effectiveness, mood, recovery

## Abstract

Stroke is frequently accompanied by long‐term sleep disruption. We therefore aimed to assess the efficacy of digital cognitive behavioural therapy for insomnia to improve sleep after stroke. A parallel group randomised controlled trial was conducted remotely in participant's homes/online. Randomisation was online with minimisation of between‐group differences in age and baseline Sleep Condition Indicator‐8 score. In total, 86 community‐dwelling stroke survivors consented, of whom 84 completed baseline assessments (39 female, mean 5.5 years post‐stroke, mean 59 years old), and were randomised to digital cognitive behavioural therapy or control (sleep hygiene information). Follow‐up was at post‐intervention (mean 75 days after baseline) and 8 weeks later. The primary outcome was self‐reported insomnia symptoms, as per the Sleep Condition Indicator‐8 (range 0–32, lower numbers indicate more severe insomnia, reliable change 7 points) at post‐intervention. There were significant improvements in Sleep Condition Indicator‐8 for digital cognitive behavioural therapy compared with control (intention‐to‐treat, digital cognitive behavioural therapy *n* = 48, control *n* = 36, 5 imputed datasets, effect of group *p* ≤ 0.02, $\eta_{p}^{2}$ = 0.07–0.12 [medium size effect], pooled mean difference = −3.35). Additionally, secondary outcomes showed shorter self‐reported sleep‐onset latencies and better mood for the digital cognitive behavioural therapy group, but no significant differences for self‐efficacy, quality of life or actigraphy‐derived sleep parameters. Cost‐effectiveness analysis found that digital cognitive behavioural therapy dominates over control (non‐significant cost savings and higher quality‐adjusted life years). No related serious adverse events were reported to the researchers. Overall, digital cognitive behavioural therapy for insomnia effectively improves sleep after stroke. Future research is needed to assess earlier stages post‐stroke, with a longer follow‐up period to determine whether it should be included as part of routine post‐stroke care. Clinicaltrials.gov NCT04272892.

## 1 INTRODUCTION

Stroke, a leading cause of disability worldwide (Johnson et al., 2019), is frequently accompanied by sleep disruption, which persists long‐term throughout recovery (Fleming et al., 2021). Insomnia, a sleep disorder characterised by difficulties initiating and maintaining sleep, is highly prevalent in this patient group (pooled prevalence estimate 32%, range 20%–70%; Baylan et al., 2020), but studies seeking to improve symptoms are scarce. As poor sleep in the stroke population is associated with depression, fatigue and reduced quality of life (Byun et al., 2019; Fleming et al., 2021; Tang et al., 2015), this presents an important area for therapeutic consideration. Additionally, sleep difficulties after acquired brain injury, including stroke, have also been correlated with poorer recovery outcomes (Fleming et al., 2020). This may be due to several different factors, including a reduced ability to engage in rehabilitation activities following a night of disrupted sleep (Worthington & Melia, 2006). Another potential factor is the importance of sleep for memory consolidation processes, whereby it is posited that during sleep, reactivation of neural activity previously learned during wake occurs, thus leading to improvements in consolidation of motor learning (Ramanathan et al., 2015). This further highlights the potential clinical significance for the need of good sleep in this patient group.

Cognitive behavioural therapy (CBT) is the first‐line recommended treatment for insomnia that has been shown to be effective in improving sleep across a range of patient groups, and preliminary efficacy for in‐person or hybrid treatment has been demonstrated following stroke (Ford et al., 2022; Herron et al., 2018; Nguyen et al., 2017). However, provision of in‐person CBT is limited by scarcity of trained therapists, long‐wait lists and is costly, culminating in high unmet demands for treatment (Koffel et al., 2018). Digital CBT (dCBT) mitigates these limitations and provides an option for delivering treatment at scale. Indeed, Sleepio (an automated dCBT programme) is effective at improving insomnia (Espie et al., 2012), mood (Luik et al., 2017) and cognitive function (Kyle et al., 2020). Although the effects of stroke, such as reduced mobility or pain, may generate potential barriers to completing standard dCBT techniques, we have previously demonstrated that with some additional information supplied to aid usability in this group (such as potential modifications to behavioural advice and screenshots to facilitate navigation within the digital programme itself), Sleepio can be feasibly used by community‐dwelling stroke survivors (Smejka et al., 2022). However, many stroke survivors also experience long‐term difficulties with movement, language and cognition, which are factors that could potentially impact the effectiveness of a behavioural intervention. As such, it is important to determine the efficacy of dCBT in this population specifically before recommending its use.

This study aimed to assess the efficacy of dCBT to improve sleep in chronic stroke survivors. We hypothesised that dCBT would result in greater improvements to sleep than provision of sleep hygiene information, and that these improvements would be sustained at least 8 weeks later. Our secondary aims were to assess the effects on mood, quality of life and self‐efficacy, as well as actigraphy‐derived sleep parameters. Finally, to evaluate the real‐world impact of improved sleep for this population, we explored the cost‐effectiveness of dCBT.

## 2 METHODS

### 2.1 Design

This was a two‐arm parallel group, randomised controlled trial comparing dCBT with provision of sleep hygiene information. The study was approved by the University of Oxford Central University Research Ethics Committee (R40803) and registered as a clinical trial prior to enrolment of the first participant (clinicaltrials.gov NCT04272892).

### 2.2 Participants

To be eligible, participants had to be: (1) aged > 18 years; (2) > 3 months post‐stroke; (3) interested in improving their sleep; (4) living in the UK with reliable internet access; (5) able to understand verbal and written English (with assistance from carer if needed); and (6) able and willing to provide informed consent. Participants were excluded if they: (1) had a serious clinical condition that could affect participation in the study, including scheduled surgery in the next 5 months; (2) were currently undergoing a psychological treatment programme for insomnia; (3) were pregnant; (4) had uncontrolled seizures (contraindication to sleep restriction included as part of dCBT); (5) had untreated diagnosed obstructive sleep apnea; or (6) did habitual shift‐work.

Participants were recruited from the community between February 2020 and June 2021, by advertising through UK stroke and brain injury charities, stroke user/support groups, social media, and our research database. After receiving the information sheet, potential participants had the opportunity to discuss the study with a researcher, including discussion of inclusion and exclusion criteria. All participants confirmed with the researcher that they met the eligibility criteria via self‐report. Participants then provided written informed consent online (using the Jisc platform). All participants (regardless of group allocation) received online shopping vouchers as compensation for their time (£15 per assessment time‐point, maximum £45).

### 2.3 Outcomes

Outcome assessments were collected at baseline, at the end of treatment (herein termed “post‐intervention”), and at a follow‐up 8 weeks later. Participants completed assessments online, except the EuroQol 5‐dimension questionnaire (EQ‐5D) and actigraphy, which were posted to their home.

The primary outcome was insomnia symptoms at the post‐intervention time‐point, which were assessed using the Sleep Condition Indicator‐8 (SCI‐8, maximum score 32). The SCI‐8 was developed to evaluate insomnia based on the 5th edition of the Diagnostic and Statistical Manual of Mental Disorders (DSM‐5), with higher values indicating fewer insomnia symptoms. The initial validation study identified that scores ≤ 16 indicate probable insomnia (Espie et al., 2014), though to increase generalisability of this study, participants with a score of > 16 were also included provided that they were interested in improving their sleep. To our knowledge, the SCI‐8 has not been specifically validated in a stroke population, but previous studies have identified significant differences in scores for people with stroke (Fleming et al., 2021) and brain injury (Fleming et al., 2020) in comparison with age‐ and sex‐matched controls.

Secondary sleep outcomes included maintenance of effects using the SCI‐8 at the 8‐week follow‐up time‐point, as well as sleep‐onset latency (SOL; from the online sleep diary recorded during the first and last week of the intervention period), and actigraphy‐derived sleep measures (estimated total sleep time, wake after sleep onset [WASO], sleep fragmentation index; Methods 1.5 in Appendix S1) at post‐intervention and at the 8‐week follow‐up. The actigraphy device used was a Motionwatch‐8 (Camntech), worn on the participant's least‐affected wrist for 7 nights per time‐point. As we opted to include participants whose sleep problems were not severe enough to be considered probable insomnia, which could limit the effect size observed, we also intended to assess SCI‐8 score post‐intervention for the sub‐sample of participants who had probable insomnia (SCI‐8 ≤ 16) at baseline (Espie et al., 2014).

Other secondary outcomes included mood, as assessed by the Patient Health Questionnaire (PHQ‐9; maximum score 27; Kroenke et al., 2001) and the Generalised Anxiety Disorder questionnaire (GAD‐7; maximum score 21; Spitzer et al., 2006); and self‐efficacy as per the Stroke Self‐Efficacy questionnaire (SSE; maximum score 130; Jones et al., 2008). Quality of life was assessed with the EQ‐5D‐5L™ (Herdman et al., 2011) and the Short‐Form‐Stroke Impact Scale (SF‐SIS; maximum index value 100; Jenkinson et al., 2013).

### 2.4 Resource use and unit costs

Participants completed a bespoke version of the Client Service Receipt Inventory (Beecham & Knapp, 2001) to categorise National Health Service (NHS) resource use over the 8 weeks prior to randomisation and over the 8‐week follow‐up period (Methods 1.1 in Appendix S1).

The per‐patient cost of Sleepio was set at £45 based on National Institute for Health and Care Excellence (NICE) guidelines. We included the following resource use categories over the 8‐week periods: hospitalisations; outpatient consultations with psychiatrists or other hospital consultants; day hospital visits; primary care visits; visits with therapists; social worker visits; and day care contacts. Outside the health and social care perspective adopted, we also asked patients to complete the nature and amount of any informal care (i.e. unpaid care from relatives, friends or neighbours) received. Unit costs were obtained from the Personal Social Services Research Unit's publication for 2020 (Curtis & Burns, 2020) and the NHS Schedule of Reference Costs for 2020.

### 2.5 Randomisation

Following baseline assessment, participants were randomised to intervention (dCBT) or control (sleep hygiene information) using freely available online software (rando.la), with minimisation of factors age and baseline SCI‐8 score to attempt to ensure balance across the two groups. MKF held access to the randomisation software, and upon completion of each participant's baseline assessment, entered their SCI‐8 score and age to perform the randomisation. Group allocation was then recorded onto a spreadsheet for the research assistant to access, and to inform the participant of their allocation (identified as Group 1 or Group 2 to the participants). Thus, the study team did not know which treatment would be assigned prior to recruitment.

Due to the nature and practicalities of the intervention, it was not possible to blind participants or the research team to group allocation. However, a blind‐to‐hypothesis approach was used whereby participants were told that the study was testing sleep improvement interventions, but not which group was anticipated to show greater effects. Those who analysed the data (MKF, RLF) had minimal interactions with participants throughout the intervention period.

### 2.6 Intervention group

Participants were given access to Sleepio (www.sleepio.com), comprising of six weekly automated online sessions (each 15–20 min) delivering evidence‐based CBT techniques for insomnia, and completion of a daily sleep diary for the intervention period (Methods 1.2 in Appendix S1). Based on feedback received during our previous qualitative usability study of Sleepio (Smejka et al., 2022), participants were also emailed a document at the beginning of the intervention period that provided additional information to aid using the programme in the context of stroke (Methods 1.2 in Appendix S1). In instances in which participants chose not to complete all CBT sessions, they were asked to complete the post‐intervention assessment as soon as possible.

### 2.7 Control group

Participants were emailed a sleep hygiene brochure containing suggestions on lifestyle and environmental factors associated with sleep disturbance (Methods 1.3 in Appendix S1), and they completed a daily online sleep diary for 1 week at the beginning and end of the intervention period (with questions matching those of the intervention group).

### 2.8 Sample size

The sample size calculation was based on the primary outcome (SCI‐8 score). We intended to collect 68 complete datasets (1:1 ratio). Based on the between‐group effect size estimate of *d* = 1.2 (Espie et al., 2012), it was initially determined that 24 participants were required (*α* = 0.05, power 80%). However, as we opted to include people with sleep difficulties who would not meet the criteria for clinical insomnia and anticipated a more modest effect of *d* = 0.7 (Smejka et al., 2022), we thus determined that 68 full datasets were required. Allowing for withdrawal, we aimed to enrol 86 participants. We anticipated this would enable a full group analysis for the primary outcome, and a secondary subgroup analysis including only participants with probable insomnia (as per the SCI‐8).

When 56 participants had been recruited, it was clear that withdrawal from the dCBT group was such that maintaining a 1:1 randomisation would lead to insufficient dCBT participants for the intended 68 complete datasets at 1:1 ratio. The remaining 30 participants were thus randomised at 2:1 (treatment:control).

### 2.9 Analysis

#### 2.9.1 Statistics

Data for the primary outcome (SCI‐8 score at the post‐intervention time‐point) were analysed using intention‐to‐treat (ITT) with multiple imputation of missing values (Methods 1.4 in Appendix S1). We conducted an analysis of covariance (ANCOVA), with the dependent variable of SCI‐8 post‐intervention, fixed factor of Group (dCBT, control), and covariates of baseline SCI‐8 and sex.

Secondary outcomes were analysed as a complete case ITT population, restricted to randomised participants for whom data were available at all time‐points. This was chosen to limit analysis to participants who engaged in the intervention to some extent, even if they did not complete the programme. Mixed ANCOVAs were used with the within‐subject factor of time (post‐intervention and 8‐week follow‐up), between‐subject factor of group, and baseline score as a covariate. When group effects were found, chi‐square tests were used to explore differences in the proportion of participants reaching the reliable or minimal clinically important difference (MCID). Two‐sided *p*‐values (significance *p* < 0.05) are reported with estimated effect sizes (partial eta‐squared: $\eta_{p}^{2}$) where appropriate. For completeness, results of ITT analyses using linear mixed effects models are provided in Table S5.

#### 2.9.2 Cost‐effectiveness

The EQ‐5D‐5L responses were converted into utility values (van Hout et al., 2012). As no deaths were reported, individual quality‐adjusted life years (QALYs) were estimated by combining utility estimates. For the cost‐effectiveness analysis, differential mean QALYs were adjusted for baseline utility, sex and age using ordinary least squares regression.

Costs were compared using a *t*‐test. For the cost‐effectiveness analysis, differential mean costs were adjusted for baseline costs, age and sex. To evaluate if dCBT was cost‐effective, an incremental analysis was carried out, with the mean cost difference between groups divided by the mean QALY difference to give the incremental cost‐effectiveness ratio (ICER). The main analysis used a healthcare perspective, but sensitivity analyses also included informal care costs. As per NICE recommendations, we judged an intervention to be cost effective if the ICER was ≤ £20,000 per QALY gained.

The non‐parametric percentile method was used for calculating the confidence interval (CI) around the ICER, using 10,000 bootstrap estimates of the mean cost and QALY differences. The cost‐effectiveness acceptability curve was used to show the probability that dCBT is cost‐effective at a threshold of £20,000 per QALY gained, and for different values of the willingness to pay for an additional QALY.

#### 2.9.3 Exploratory mediation analysis

To further understand the effects of dCBT for insomnia on mood and actigraphy‐derived sleep disruption measures, we explored whether differences in these secondary outcomes were mediated by SCI‐8 score changes using the “mediation” package in R (Methods 1.6 in Appendix S1).

## 3 RESULTS

### 3.1 Demographics

Recruitment took place between February 2020 and June 2021, with a total of 86 participants consented as planned. The trial ended in December 2021, once all follow‐up assessments were completed. Two participants withdrew without completing the baseline assessment (no reason given). The remaining 84 participants (mean [SD] age 58.6 [13.5] years, 39 female, mean [SD] 5.5 [5] years post‐stroke) were randomised (Table 1). Based on the SCI‐8, 83% reported having sleep problems for > 6 months.

**TABLE 1:** Baseline demographics and assessment values

	dCBT	Control
*N*	48	36
Age, years	58.5 (12.7)	58.7 (14.7)
Sex (female): *N* (%)	17 (35.4%)	22 (61.1%)
Years since most recent stroke	6 (5)	6 (5)
Number of strokes, *N* (%)		
1	41 (85%)	30 (83%)
> 1	7 (15%)	6 (17%)
Relevant medications		
Antidepressant		
SSRI	8 (17%)	3 (8%)
SNRI	2 (4%)	2 (6%)
SARI	1 (2%)	0 (%)
TCA	2 (4%)	3 (8%)
Other	1 (2%)	1 (3%)
Z‐drug	0 (0%)	3 (8%)
Gabapentinoid	8 (17%)	5 (14%)
Melatonin receptor agonist	1 (2%)	1 (3%)
Patient‐reported outcomes		
*N*	48	36
SCI‐8	12.0 (6.3)	11.4 (7.0)
*Probable insomnia*: *N* (%)	38 (79.2%)	30 (83.3%)
PHQ‐9	10.2 (4.4)	9.5 (5.1)
GAD‐7	8.9 (5.5)	7.6 (5.4)
SF‐SIS	47.3 (13.2)	43.1 (13.7)
SSE	85.8 (28.6)	73.5 (36.5)
Actigraphy		
*N*	48	34
Estimated total sleep time (hr:min)	7:01 (1:08)	7:26 (1:22)
WASO (min)	57 (28)	63 (34)
Sleep fragmentation index	31.5 (13.8)	32.1 (15.7)

*Note*: Values are mean (standard deviation) unless otherwise specified.

Abbreviation: dCBT, digital cognitive behavioural therapy; GAD‐7, Generalised Anxiety Disorder questionnaire; PHQ‐9, Patient Health Questionnaire; SARI, serotonin antagonist and reuptake inhibitor; SCI‐8, Sleep Condition Indicator‐8; SF‐SIS, Short‐Form‐Stroke Impact Scale; SNRI, serotonin and norepinephrine reuptake inhibitor; SSE, Stroke Self‐Efficacy questionnaire; SSRI, selective serotonin reuptake inhibitor; TCA, tricyclic antidepressant; WASO, wake after sleep onset; Z‐drug, e.g. Zopiclone or Zolpidem.

**FIGURE 1:**
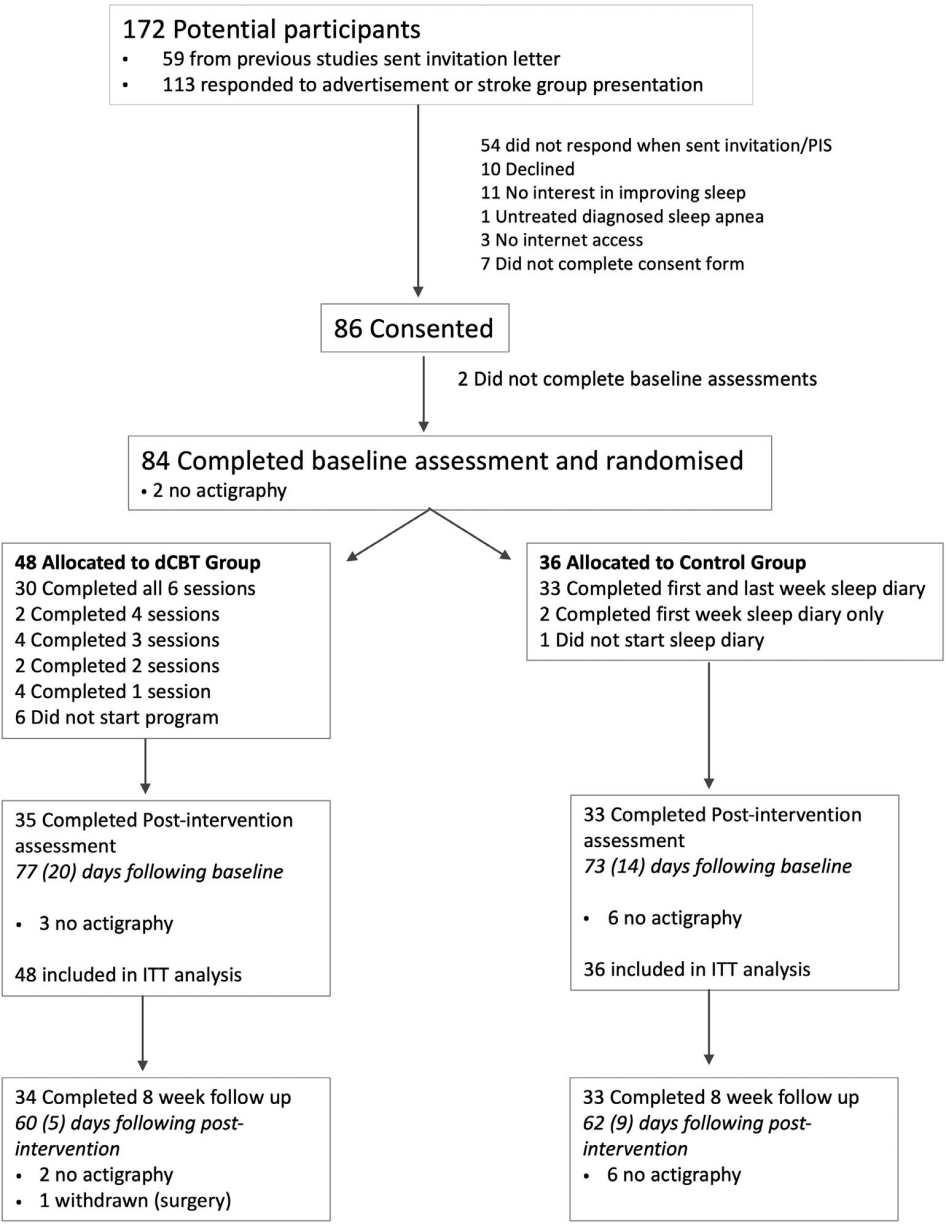
Consort diagram. For the primary outcome (SCI‐8 score post‐intervention), intention‐to‐treat (ITT) analysis was with imputation of missing data. For secondary outcomes, complete case analysis was used. dCBT, digital cognitive behavioural therapy for insomnia; PIS, participant information sheet. Mean (standard deviation) days between assessments did not differ between groups

Sixteen participants (13 dCBT, three control) withdrew without completing the post‐intervention assessment (Figure 1; Table S1). Comparison of characteristics between participants who withdrew and those who completed the post‐intervention assessment showed that there was no difference for baseline SCI‐8 score (|*t*|[82] = 0.16, *p* = 0.874) or age (|*t*|[82] = 0.17, *p* = 0.869). However, participants who withdrew had significantly worse symptoms of depression (PHQ‐9; |*t*|[82] = 2.46, *p* = 0.016) and anxiety (GAD‐7; |*t*|[82] = 2.25, *p* = 0.027) at baseline than those who completed the study, and lower self‐efficacy (SSE; |*t*|[82] = 2.84, *p* = 0.006).

We estimated it would take 6–8 weeks for participants to complete the dCBT programme, and attempted to match control group assessment timing on an ongoing basis. Although the time from baseline to post‐intervention was on average longer than anticipated (Figure 1), timeframes did not differ between groups (*p* > 0.2).

### 3.2 Sleep condition indicator

The primary outcome, SCI‐8 score post‐intervention (ITT dCBT *n* = 48, control *n* = 36), was significantly greater following dCBT than control, adjusted for baseline SCI‐8 and sex, across original and imputed datasets (Tables 2 and S2), with a medium effect size. This is indicative of fewer symptoms of insomnia following dCBT.

**TABLE 2:** ITT analysis of primary outcome (SCI‐8 post‐intervention)

	Control		dCBT		Adjusted mean difference^a^ (95% CI)	Effect of group	Effect size ($\eta_{p}^{2}$)
	Baseline	Post‐intervention	Baseline	Post‐intervention			
Original data	11.4 (7.0)	14.9 (7.3)	12.0 (6.3)	18.5 (5.9)	−3.661 (−6.14, −1.18)	*F*_1,64_ = 8.72, *p* = 0.004	0.12
Imputation 1	–	14.7 (7.0)	–	18.2 (6.0)	−3.40 (−5.64, −1.16)	*F*_1,80_ = 9.09, *p* = 0.003	0.10
Imputation 2	–	14.6 (7.1)	–	18.5 (6.7)	−3.65 (−5.91, −1.38)	*F*_1,80_ = 10.27, *p* = 0.002	0.11
Imputation 3	–	14.8 (7.2)	–	17.6 (6.7)	−2.77 (−5.09, −0.46)	*F*_1,80_ = 5.67, *p* = 0.020	0.07
Imputation 4	–	14.5 (7.1)	–	18.3 (5.7)	−3.65 (−5.92, −1.37)	*F*_1,80_ = 10.19, *p* = 0.002	0.11
Imputation 5	–	14.6 (7.2)	–	18.1 (6.1)	−3.26 (−5.48, −1.05)	*F*_1,80_ = 8.60, *p* = 0.004	0.10
Pooled		14.6		18.1	−3.35	–	–

*Note*: Data in control and dCBT columns are non‐adjusted means and standard deviations. See Table S2 for estimated marginal means, adjusted for baseline SCI‐8/sex.

Abbreviation: CI, confidence interval; dCBT, digital cognitive behavioural therapy.

^a^ Mean difference is adjusted for baseline SCI‐8/sex. No data were imputed at baseline.

**FIGURE 2:**
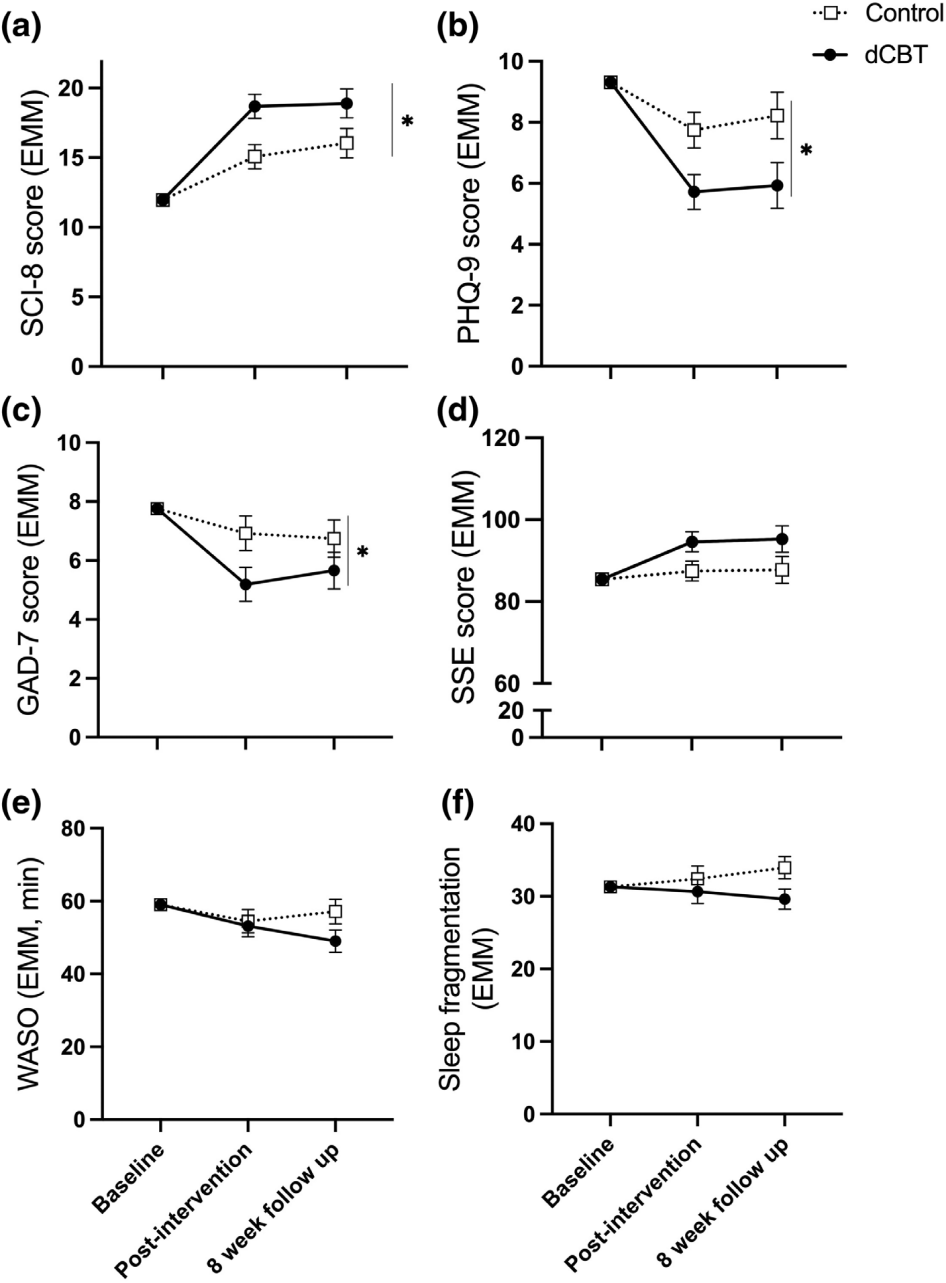
Estimated marginal means (baseline score covaried) at post‐intervention and 8‐week follow‐up for questionnaire and actigraphy measures. (a) Sleep Condition Indicator‐8 (SCI‐8; higher values indicate fewer symptoms of insomnia), (b) Patient Health Questionnaire (PHQ‐9; lower scores indicate fewer symptoms of depression), (c) Generalised Anxiety Disorder questionnaire (GAD‐7; lower scores indicate fewer symptoms of anxiety), (d) Stroke Self‐Efficacy scale (SSE; higher scores indicate better self‐efficacy), (e) wake after sleep onset (WASO; higher time is more wakefulness during sleep period), (f) sleep fragmentation index (higher values indicate more disrupted sleep). Error bars are standard error of the mean. *Significant effect of group (adjusted means were better for digital cognitive behavioural therapy [dCBT] than control, *p* < 0.05)

Our secondary objective in relation to the SCI‐8 was to test for maintenance of effects. There was an effect of group (mixed ANCOVA: *F*_1,64_ = 6.35, *p* = 0.014, $\eta_{p}^{2}$ = 0.09, medium effect), and no group by time interaction (*F*_1,64_ = 0.74, *p* = 0.39), suggesting improved SCI‐8 score for dCBT across post‐intervention and the 8‐week follow‐up time‐points (Figure 2a).

For the secondary subgroup analyses including only participants with probable insomnia, based on the SCI‐8 score at baseline (SCI‐8 ≤ 16; dCBT *n* = 29, control *n* = 27), there was an effect of group (*F*_1,52_ = 8.27, *p* = 0.006, $\eta_{p}^{2}$ = 0.14, medium effect), as adjusted SCI‐8 was higher for dCBT than control. As an additional post hoc analysis, we also found that a larger proportion of the dCBT group (baseline ≤ 16) scored > 16 at post‐intervention, suggesting symptom resolution (71% versus 30%, *χ* ^2^[1] = 9.61, *p* = 0.002). For visualisation purposes, the proportions of participants reaching the criteria for probable insomnia at each time‐point are in Figure S1.

### 3.3 Secondary patient‐reported outcomes

Group means can be found in Tables S3–S8. For depression (PHQ‐9) and anxiety (GAD‐7), there were significant group effects (PHQ‐9: *F*_1,64_ = 6.754, *p* = 0.012, $\eta_{p}^{2}$ = 0.095, medium effect; GAD‐7: *F*_1,64_ = 4.109, *p* = 0.047, $\eta_{p}^{2}$ = 0.060, medium effect), and no group by time interactions (PHQ‐9: *F*_1,64_ = 0.079, *p* = 0.780; GAD‐7: *F*_1,64_ = 0.405, *p* = 0.527), suggesting improved mood following dCBT compared with control across follow‐up time‐points (Figure 2b,c).

For SOL, taken from the online sleep diary, the ANCOVA (with first week as covariate) showed a significantly shorter sleep latency for dCBT than control at the end of the intervention period (*F*_1,63_ = 6.406, *p* = 0.014, $\eta_{p}^{2}$ = 0.092, medium effect).

For self‐efficacy (SSE) there was a non‐significant effect of group (*F*_1,64_ = 3.990, *p* = 0.050, $\eta_{p}^{2}$ = 0.059, medium effect; Figure 2d), and no group by time interaction (*F*_1,64_ = 0.009, *p* = 0.923).

For quality of life, there was no effect of group for SF‐SIS (*F*_1,64_ = 0.132, *p* = 0.718), or group by time interaction (*F*_1,64_ = 0.827, *p* = 0.367). Similarly, there were no between‐group differences for EQ‐5D utilities or visual analogue scale scores (see Table S6 for adjusted mean differences; *p* > 0.05).

### 3.4 Costs and cost‐effectiveness

Over the 8‐week follow‐up period, and after including the costs of Sleepio, the dCBT group had non‐significantly lower NHS care costs than control (adjusted mean difference −£349, 95% CI: −1035 to 337, *p* = 0.31; Table S9). After inclusion of informal care costs, the adjusted mean cost difference was −£330 (95% CI: −1550 to 891, *p* = 0.59).

From a healthcare perspective, dCBT was dominant over sleep hygiene information (i.e. overall cost savings and associated with higher QALYs; Table 3). The probability that dCBT was cost‐saving was 0.876. The probability increased to 0.885 and 0.911 at £20,000 and £100,000 per QALY gained threshold, respectively.

**TABLE 3:** Cost‐effectiveness of dCBT (treatment) compared with control

	Control	dCBT	Adjusted mean difference^a^
	Mean (SD)	Mean (SD)	95% CI
NHS costs	639 (1882)	191 (392)	−349 (−1035 to 337)
QALYs	0.0137 (0.003)	0.0141 (0.003)	0.0003 (−0.001 to 0.001)
ICER – NHS			Treatment dominates

Abbreviation: CI, confidence interval; dCBT, digital cognitive behavioural therapy; ICER, incremental cost‐effectiveness ratio; NHS, National Health Service; QALYs, quality‐adjusted life years.

^a^ Adjusted for baseline cost/utility, age and sex.

### 3.5 Secondary actigraphy outcomes

There were no group effects (all *F*_1,54_ < 2.6, *p* > 0.1), or group by time interactions (all *F*_1,54_ < 2.8, *p* > 0.09) for any of the analysed actigraphy parameters (Figure 2e,f; Table S3).

### 3.6 Estimate of importance of difference

For outcomes where a significant group effect was observed, we assessed the proportion of participants meeting the reliable or MCID. There is no established MCID for SCI‐8, but more dCBT participants experienced a reliable change at post‐intervention (≥ 7 points [Espie et al., 2018], 49% versus 21%, *χ* ^2^[1] = 5.57, *p* = 0.018), which was not statistically significant at the 8‐week follow‐up (50% versus 30%, *χ* ^2^[1] = 2.70, *p* = 0.100).

Similarly, significantly more dCBT participants reached the MCID for PHQ‐9 and GAD‐7 at post‐intervention (PHQ‐9: ≥ 5 points [Kroenke et al., 2001], 40% versus 15%, *χ* ^2^[1] = 5.21, *p* = 0.022; GAD‐7: ≥ 4 points [Toussaint et al., 2020], 43% versus 12%, *χ* ^2^[1] = 7.97, *p* = 0.005), but this was not significant at the 8‐week follow‐up (PHQ‐9: 32% versus 15%, *χ* ^2^[1] = 2.73, *p* = 0.099; GAD‐7: 35% versus 15%, *χ* ^2^[1] = 3.588, *p* = 0.058).

### 3.7 Exploratory mediation analyses

**FIGURE 3:**
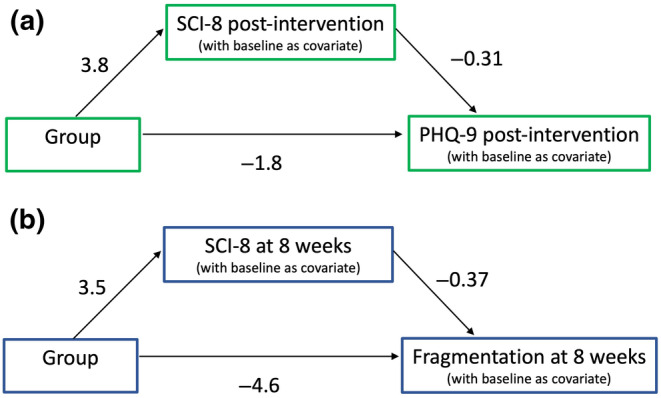
Exploratory mediation analyses. (a) Sleep Condition Indicator‐8 (SCI‐8) mediates the effect of group on Patient Health Questionnaire (PHQ‐9). The average causal mediation effect (ACME) was −1.18 (*n* = 68). (b) The ACME for sleep fragmentation was −1.24 (*n* = 59)

The effect of group on PHQ‐9 at post‐intervention was mediated via the SCI‐8 score (Figure 3a). The average causal mediation effect (ACME) was −1.18 (95% CI: −2.25 to −0.37), *p* = 0.002. This suggests that reductions in depression are mediated by improvements in insomnia symptoms. No mediation effects were observed for GAD‐7 (ACME −0.33 [95% CI: −1.06 to 0.30], *p* = 0.256).

Finally, given the (non‐significant) visual tendency for lower WASO and sleep fragmentation for dCBT at the 8‐week follow‐up (Figure 2e,f), we explored whether SCI‐8 mediated apparent changes in sleep disruption from actigraphy. The ACME was −1.47 [95% CI: −5.04 to 1.26], *p* = 0.264 for WASO and −1.24 [95% CI: −3.26 to 0.01], *p* = 0.054 for sleep fragmentation.

### 3.8 Adverse events

No related serious adverse events were reported to the research team. Other adverse effects related to study participation are in the Results 2.5 in Appendix S1.

## 4 DISCUSSION

This study found significantly fewer symptoms of insomnia for community‐dwelling stroke survivors following dCBT for insomnia compared with provision of sleep hygiene information. dCBT for insomnia, specifically Sleepio, is effective across a range of clinical populations, and this is the first study to demonstrate efficacy after stroke. Results of the cost‐effectiveness analyses also suggest that dCBT has the potential to be cost effective in this population.

Insomnia symptoms are highly prevalent after stroke (Baylan et al., 2020), and poor sleep is also a risk factor for stroke (Gottlieb et al., 2019). Here we demonstrate improvements in SCI‐8 score following dCBT, with an overall medium effect size across ITT and complete case analyses. Participants seeking to improve sleep were included, regardless of severity, to increase generalisability. This may have limited the magnitude of our effect, but previous research indicates some efficacy of dCBT in treating sub‐threshold insomnia symptoms (Denis et al., 2020). Our sub‐analysis restricted to participants with probable insomnia (based on baseline SCI‐8 score) demonstrated similar findings to the full group analysis. We found a reliable change for approximately half of the dCBT group who completed the follow‐up assessments. This is broadly comparable with the proportions of clinically meaningful improvements found previously with in‐person or hybrid treatments following acquired brain injury (Ford et al., 2022; Ymer et al., 2021). There is growing evidence that improvements conferred by CBT are sustained for at least 6–12 months (Luik et al., 2020; van der Zweerde et al., 2019). Nevertheless, given the long‐term nature of stroke, future studies with a longer follow‐up are needed to ascertain whether repeat or refresher sessions will be required to prevent return of sleep problems. Additionally, as we deliberately included community dwelling, rather than hospitalised, stroke survivors, it is not anticipated that the findings translate directly to the early stages of stroke recovery. Further studies are needed to evaluate and implement evidence‐based treatments for sleep in acute and rehabilitation stroke units.

Mood disorders are highly prevalent after stroke (Jørgensen et al., 2016; Rafsten et al., 2018), and we found fewer symptoms of depression and anxiety following dCBT for insomnia. Our exploratory mediation analysis suggests that improvements in depression are mediated to some extent by improvements in SCI‐8, as seen in adults without stroke (Henry et al., 2021). Sleep may therefore serve as a treatment target to improve mental health in this population. However, it is important to consider the inherent relationships between self‐reported measures, for example individuals low in mood or confidence may rate their sleep as worse. This is particularly relevant given that there were no significant actigraphy changes. We must also consider the potential that treatments for mood disorders may also impact on sleep, and while details of self‐report anti‐depressant medications were collected (Table 1), we did not conduct any statistical analyses on these due to the low numbers reported. Additionally, we did not collect information on whether participants were receiving any form of psychological therapy for depression or anxiety, in which sleep difficulties may have been addressed to some extent. Although sleep components tend to make up only a small part of such interventions, we cannot rule out the potential presence of these therapies or the possibility that they may have influenced the changes in sleep observed.

A recent meta‐analysis demonstrated that there are typically minimal or no improvements in actigraphy or polysomnography outcomes following CBT (Mitchell et al., 2019). Although reasons for this are unclear, it may be partly explained by studies recruiting via self‐reported rather than objective measures, as insomnia is defined by self‐reported complaints. For the current study, actigraphy data were not available from all participants (reasons in Results 2.6 in Appendix S1), and although no statistical significance was found there is some apparent visual tendency towards improved sleep fragmentation index and WASO at 8 weeks following dCBT (Figure 2). It may be that improvements in actigraphy parameters could develop after initial changes in sleep habits and perception in this patient group. However, this is entirely speculative and requires adequately powered studies with a longer follow‐up to investigate. It would also be interesting for future studies to ask participants to wear the actigraphy monitor during the intervention period, to better understand fluctuations in actigraphy variables throughout the process. Nonetheless, our exploratory analyses are encouraging, suggestive of a tendency towards less sleep fragmentation with improvements in SCI‐8.

## 5 LIMITATIONS

Despite conducting a qualitative study to understand and address usability concerns (Smejka et al., 2022), a substantial proportion of participants still withdrew. The dropout was comparable to that found previously in people without stroke (Espie et al., 2012; Ho et al., 2015; Seyffert et al., 2016). Withdrawn participants exhibited lower mood and self‐efficacy at baseline, which is consistent with studies from other populations using in‐person and dCBT (Ong et al., 2008; Yeung et al., 2015). Because active engagement is required, first addressing feelings of low mood and confidence may be beneficial. A hybrid model, whereby dCBT is combined with clinician input, may help participants to discuss options and receive support for self‐managing treatment. Indeed, hybrid treatment was effective in a sample of 52 people with stroke or traumatic brain injury (Ford et al., 2022).

We did not employ a systematic method for obtaining information relating to adverse effects, instead relying on participants to inform us if they had any concerns. We therefore cannot rule out the possibility that there were adverse events or side‐effects that we were not made aware of. A recent systematic review highlighted that future studies need improved prospective monitoring and reporting to ensure that side‐effects are better understood (Condon et al., 2021).

We used ITT analysis for the primary outcome but chose to use complete case analyses for the secondary measures. We acknowledge that this introduces bias, but felt it important to examine changes in secondary outcomes specifically for stroke survivors who were able and willing to engage in the intervention to some extent. The current study therefore provides an initial assessment of outcomes that may or may not be responsive to dCBT in this population, but researchers in the future should use these results to guide study design to attempt replication.

As we did not have access to brain imaging or medical records, we were unable to obtain details of the participants' strokes and relied on them to self‐report that they had received a stroke diagnosis and the date on which the stroke occurred. It is unknown whether stroke lesion characteristics impacted on sleep or treatment response, or whether stroke characteristics differed between groups. The effect observed here may also be limited by undiagnosed comorbid sleep disorders, for example sleep apnea, although CBT for insomnia remains effective in people with comorbid obstructive sleep apnea (Sweetman et al., 2017). We were unable to complete in‐person sleep assessments and relied on participant report for diagnoses of sleep apnea. Though we acknowledge these are clear limitations, we nevertheless demonstrate significant sleep improvements in this cohort.

Results of the cost‐effectiveness analyses should be interpreted cautiously given the relatively small sample and short follow‐up duration. Nevertheless, the promising results are consistent with previous studies demonstrating cost‐effectiveness (Darden et al., 2021), and should be extended in a larger trial.

## 6 CONCLUSION

Cognitive behavioural therapy is the first‐line recommendation for treatment of insomnia, and here we provide evidence of efficacy of dCBT in community‐dwelling stroke survivors. More research is needed to ascertain who is most likely to benefit, the extent to which efficacy is similar earlier after discharge from hospital, and how long effects persist.

## AUTHOR CONTRIBUTIONS

**Melanie K. Fleming:** Conceptualization; data curation; formal analysis; investigation; methodology; project administration; supervision; writing – original draft; writing – review and editing. **Tom Smejka:** Conceptualization; data curation; investigation; methodology; project administration; writing – review and editing. **Ellie Macey:** Data curation; formal analysis; investigation; methodology; project administration; writing – review and editing. **Ramon Luengo‐Fernandez:** Data curation; methodology; visualization; writing – original draft; writing – review and editing. **Alasdair L. Henry:** Conceptualization; methodology; resources; writing – review and editing. **Barbara Robinson:** Formal analysis; writing – review and editing. **Simon D. Kyle:** Conceptualization; methodology; writing – review and editing. **Colin A. Espie:** Conceptualization; resources; writing – review and editing. **Heidi Johansen‐Berg:** Conceptualization; funding acquisition; supervision; writing – review and editing.

## FUNDING INFORMATION

This study is funded by the Wellcome Trust (110027/Z/15/Z) and supported by the NIHR Oxford Biomedical Research Centre. The Wellcome Centre for Integrative Neuroimaging is supported by core funding from the Wellcome Trust 203139/Z/16/Z.

## CONFLICT OF INTEREST STATEMENT

CAE is co‐founder and Chief Scientist of Big Health Ltd, is salaried and is a shareholder. ALH is employed by Big Health Ltd, is salaried and a shareholder. Big Health Ltd supplied Sleepio free of charge. All other authors report no conflicting interests.

## Supporting information


Appendix S1.

## Data Availability

The data that support the findings of this study are openly available on the Open Science Framework at https://osf.io/zmvfx/.
